# Lysosome assembly and disassembly changes endocytosis rate through the *Leishmania* cell cycle

**DOI:** 10.1002/mbo3.969

**Published:** 2019-11-19

**Authors:** Ziyin Wang, Richard J. Wheeler, Jack D. Sunter

**Affiliations:** ^1^ Sir William Dunn School of Pathology University of Oxford Oxford UK; ^2^ The Peter Medawar Building for Pathogen Research University of Oxford Oxford UK; ^3^ Department of Biological and Medical Sciences Oxford Brookes University Oxford UK

**Keywords:** cell cycle, endocytosis, *Leishmania*, lysosome

## Abstract

The *Leishmania* lysosome has an atypical structure, consisting of an elongated vesicle‐filled tubule running along the anterior–posterior axis of the cell, which is termed the multivesicular tubule (MVT) lysosome. Alongside, the MVT lysosome is one or more microtubules, the lysosomal microtubule(s). Previous work indicated there were cell cycle‐related changes in MVT lysosome organization; however, these only provided snapshots and did not connect the changes in the lysosomal microtubule(s) or lysosomal function. Using mNeonGreen tagged cysteine peptidase A and SPEF1 as markers of the MVT lysosome and lysosomal microtubule(s), we examined the dynamics of these structures through the cell cycle. Both the MVT lysosome and lysosomal microtubule(s) elongated at the beginning of the cell cycle before plateauing and then disassembling in late G_2_ before cytokinesis. Moreover, the endocytic rate in cells where the MVT lysosome and lysosomal microtubule(s) had disassembled was extremely low. The dynamic nature of the MVT lysosome and lysosomal microtubule(s) parallels that of the *Trypanosoma cruzi* cytostome/cytopharynx, which also has a similar membrane tubule structure with associated microtubules. As the cytostome/cytopharynx is an ancestral feature of the kinetoplastids, this suggests that the *Leishmania* MVT lysosome and lysosomal microtubule(s) are a reduced cytostome/cytopharynx‐like feature.

## Graphical abstract

The Leishmania lysosome has an atypical structure, consisting of a vesicle‐filled tubule called the multivesicular tubule (MVT) lysosome. We examined the dynamics of this structure through the cell cycle using mNeonGreen tagged protein markers; the MVT lysosome elongated during the cell cycle and then disassembled before cytokinesis. The disassembly of the MVT lysosome coincided with a reduction in endocytosis rate.

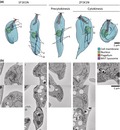

## 1 INTRODUCTION

The kinetoplastids are flagellated single‐celled eukaryotes whose shape and form are defined by a regular array of subpellicular microtubules. They have a single flagellum that extends from the flagellar pocket, which is an invagination of the cell body membrane and the site of the trafficking of macromolecular material into and out of these organisms (Field & Carrington, 2009). Many kinetoplastid parasites, including *Leishmania* and *Trypanosoma cruzi*, also have additional microtubules within the cytoplasm with one end of these microtubules normally positioned in close proximity to the flagellar pocket (Alcantara, Vidal, Souza, & Cunha‐e‐Silva, 2014; Lacomble et al., 2009; Wheeler, Sunter, & Gull, 2016). For kinetoplastid cell forms with the flagellum laterally attached to the side of the cell body, such as the *Trypanosoma brucei* trypomastigote, the subpellicular microtubule array is interrupted by a specialized set of four microtubules called the microtubule quartet (MtQ) that forms part of the flagellum attachment zone (Lacomble et al., 2009; Sunter & Gull, 2016; Vidal & Souza, 2017). The flagellum attachment zone MtQ is nucleated close to the base of the flagellar pocket and then wraps around the pocket before invading the subpellicular array, following the line of flagellum attachment (Lacomble et al., 2009). In the *Leishmania* promastigote form, which does not have lateral attachment of the flagellum to the cell body, the flagellum attachment zone MtQ is present around the flagellar pocket and does not invade the subpellicular microtubule array (Wheeler et al., 2016).

The terminal endocytic compartment in *Leishmania*, the multivesicular tubule (MVT) lysosome, has a structure atypical of a lysosome. The MVT lysosome comprises a long vesicle‐filled tubule that stretches from the flagellar pocket region at the cell anterior beyond the nucleus into the posterior end of the cell and is associated with one or two microtubules (Mullin et al., 2001; Waller & McConville, 2002; Weise, Stierhof, Kuhn, Wiese, & Overath, 2000). A low pH is important for the maintenance of this elongated tubular structure as the addition of bafilomycin A_1_, a specific inhibitor of vacuolar‐type H^+^ ATPases, caused a rapid collapse in the MVT lysosome (Mullin et al., 2001). The MVT lysosome contains cysteine and serine proteases as expected for a degradative compartment; however, the pH of this organelle appears less acidic than typical lysosomes as it is not readily stained with lysotracker, which accumulates in low pH organelles (Besteiro, Williams, Coombs, & Mottram, 2007; Mullin et al., 2001). The MVT lysosome was identified by Ilgoutz and colleagues using BODIPY‐C_5_‐Cer and a GFP tagged dolichol‐phosphate‐mannose synthase (DPMS) and was initially called the DPMS tubule (Ilgoutz, Mullin, Southwell, & McConville, 1999). Subsequent work by Weise and colleagues showed that this DPMS tubule was likely to be a lysosomal compartment; this was confirmed through further work by Mullin and colleagues who showed by immunoelectron microscopy that DPMS localized to the MVT lysosome (Mullin et al., 2001; Weise et al., 2000).

The lysosome in *T. brucei* does not have the elongated tubule structure observed in *Leishmania* and instead forms a rounded vesicular structure on the posterior side of the nucleus (Halliday et al., 2019; Langreth & Balber, 1975; Peck et al., 2008). The presence of a lysosome in *T. cruzi* has been the subject of debate: The terminal endocytic compartment was initially termed a reservosome as the structure lacked acid phosphatase activity and was not labeled with antibodies that recognize mammalian lysosome membrane proteins (Soares, Souto‐Padrón, & Souza, 1992). Further work has shown that there are generally multiple reservosomes in a cell, which are spherical membrane‐bound structures found in the posterior end of the cell with characteristics of prelysosomes, lysosomes, and recycling compartments, and have now been classified as lysosomal‐related organelles (Cunha‐e‐Silva et al., 2006; Sant’Anna et al., 2008).

*Trypanosoma cruzi* has an additional endocytic organelle, the cytostome/cytopharynx, which is a long membrane tube that invades deep into the cell body with the entrance positioned close to the flagellar pocket. The cytostome/cytopharynx is the major route for bulk endocytosis into this parasite, and this structure is not found in *Leishmania* and *T. brucei,* but was likely present in the ancestral kinetoplastid (Skalický et al., 2017). There are two sets of microtubules, one a microtubule triplet and the other a microtubule quartet (distinct from the flagellum attachment zone MtQ) associated with the cytostome/cytopharynx complex. The cytostome/cytopharynx microtubule quartet is nucleated near the flagellar pocket and then extends out beyond the pocket, just under the cell membrane along the preoral ridge before dropping into the cytoplasm alongside the cytostome/cytopharynx. Conversely, the microtubule triplet is nucleated near the cytostome/cytopharynx entrance, and together, these two sets of microtubules form a V shape upon which the cytostome/cytopharynx sits (Alcantara et al., 2014). In the latter stages of the cell cycle, during G_2_ prior to flagellar pocket division, the cytostome/cytopharynx complex and associated microtubules are disassembled, and then, the structure reassembles during late cytokinesis (Alcantara, L., Vidal, J.C., Souza, W. de, & Cunha‐e‐Silva, N.L., 2017). Interestingly, it has also been shown that the MVT lysosome in dividing *Leishmania* cells also disassembles forming one or two sets of vesicles (Ilgoutz et al., 1999; Weise et al., 2000).

Here, we used cysteine peptidase A (CPA) and sperm flagellar 1 (SPEF1) as markers of the MVT lysosome and its associated microtubule, respectively, to characterize the cell cycle‐related changes in these structures. We show that both the lysosome and its microtubule extend during G_1_/S phase of the cell but disassemble rapidly during G_2_ and are essentially absent during cytokinesis before assembling again during the next G_1_. This cycle of assembly and disassembly is associated with a change in the endocytic capacity of the *Leishmania* cell.

## 2 RESULTS AND DISCUSSION

### 2.1 MVT lysosome disassembles prior to cell division

We have previously identified cysteine peptidase A (CPA) as a MVT lysosomal protein amenable to analysis by microscopy when tagged with a fluorescent protein (Halliday et al., 2019). We also determined that SPEF1, a protein originally identified as a proximal flagellum attachment zone MtQ‐associated protein in *T. brucei* (Gheiratmand, Brasseur, Zhou, & He, 2013), localizes to an additional structure in *L. mexicana* which could plausibly be the lysosomal microtubule(s; Halliday et al., 2019). Previous work by other groups had indicated that there were cell cycle‐related changes in the organization of the MVT lysosome (Ilgoutz et al., 1999; Weise et al., 2000). However, these studies had limited time resolution and only provided snapshots of the MVT lysosome and did not link these changes in MVT lysosome organization to the lysosomal microtubule(s) or lysosomal function. We wanted to examine these organizational changes, the interrelationships between them, and their functional consequences in detail using direct markers of these structures.

**Figure 1:**
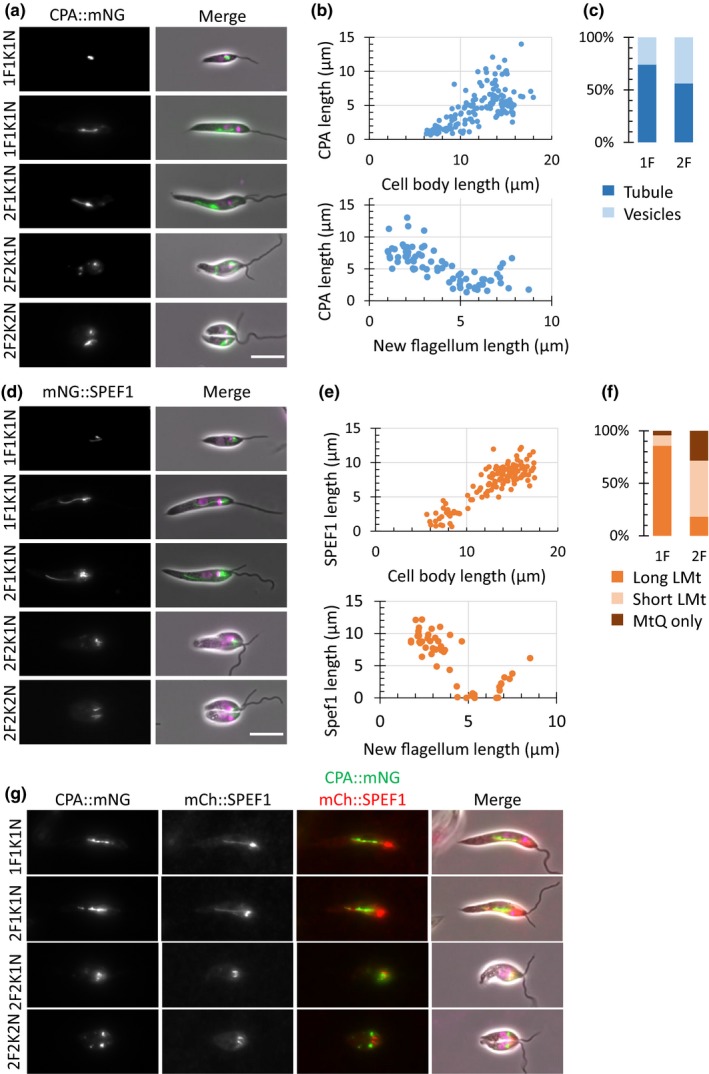
Localization and morphological changes in CPA::mNG and mNG::SPEF1 throughout the cell cycle. Images of CPA::mNG (a) or mNG::SPEF1 (d) localization during the cell cycle in the *Leishmania* promastigote. Micrographs of major cell cycle stages, cells were ordered based on the number of kinetoplasts (K), nuclei (N), and flagella (F). Nuclear and kinetoplast DNA were labeled with Hoechst 33342. The scale bar represents 5 µm. Scatter plot of cell body length or new flagellum length (measured from cell tip to flagellum tip) against CPA::mNG (B) or mNG::SPEF1 (e) length in 1F or 2F stage, respectively. Each dot represents one cell, *n* = 303 for 1F and 101 for 2F CPA::mNG, *n* = 297 for 1F and 98 for 2F mNG::SPEF1. Bar charts of CPA::mNG (c) or mNG::SPEF1 (f) signal categories in 1F and 2F cells. (g) Micrographs of CPA:: mNG (green) mCh::SPEF1 (red) and colocalization during the cell cycle in the *Leishmania* promastigote. MtQ—microtubule quartet; LMt—lysosomal microtubule(s)

We generated cell lines expressing CPA and SPEF1 tagged with mNeonGreen (mNG) at their endogenous loci and examined their localization during the cell cycle (Figure 1a,d). The *Leishmania* cell cycle stage can be determined by examining specific cellular features including the number of kinetoplasts (mitochondrial DNA‐containing structures), nuclei, and flagella present in a cell as these structures duplicate at specific points during the cell cycle. At the start of the cell cycle, a cell has one flagellum, one kinetoplast, and one nucleus (1F1K1N), and in this cell type, CPA::mNG had two distinct types of localization. CPA::mNG either localized to a number of vesicles distributed in the cytoplasm, which were normally concentrated at the anterior end of the cell or to an elongated tubule that lies on the anterior–posterior axis of the cell, extending from close to the kinetoplast before curving around the nucleus and terminating in the posterior half of the cell. We quantified this distribution and found 74% of cells had an elongated tubule signal, and the rest had a vesicular signal. During the *Leishmania* cell cycle, there is an initial increase in cell body length, which then plateaus before the new flagellum begins to extend and the cells then shorten during cytokinesis (Wheeler, Gluenz, & Gull, 2011). We measured cell body length and the length of the CPA::mNG labeled lysosome in 1F1K1N cells (Figure 1b). There was a positive correlation between cell body length and CPA::mNG signal length, with CPA::mNG signal length increasing as cell body length increased. Moreover, the shorter cells at the start of the cell cycle tended to have the vesicular CPA::mNG signal, and this suggests that as the cell cycle progressed the lysosome switched from a vesicular to an extended tubular structure.

When we examined cells with two flagella, we again observed that cells either had a tubular (56%) or vesicular (44%) CPA::mNG signal (Figure 1c). To determine the cell cycle position of the cells with two flagella, we measured the length of the new flagellum and correlated that with the length of the CPA::mNG signal (Figure 1b). In these cells, the CPA::mNG signal length remained relatively constant until the new flagellum reached ~4 µm long at which point the CPA::mNG signal began to shorten from the posterior end and disassembled into vesicles with no long tubule observed (Figure 1a). The disassembly correlated with the onset of cell division, and our images appeared similar to a dividing cell expressing GFP::DPMS (Ilgoutz et al., 1999). In the few cells in which the new flagellum was longer than ~7 µm, there was accumulation of CPA::mNG, causing an increase in the length of the CPA::mNG signal; however, this signal did not have a clear tubular structure. The increase in CPA::mNG is potentially an early step in the reassembly of the MVT lysosome.

In *L. mexicana,* mNG::SPEF1 localized to structures around the flagellar pocket, including the MtQ and possibly the pocket microtubules. In addition, mNG::SPEF1 localized to a linear structure that extended from close to the kinetoplast, curving around the nucleus before terminating in the posterior half of the cell (Figure 1d), similar to the localization of CPA::mNG. SPEF1 is likely a binding lysosomal microtubule or microtubules. We determined whether the lysosomal microtubule(s) have cell cycle dynamics similar to the MVT lysosome. We quantified the mNG::SPEF1 signal type in cells with both one or two flagella (Figure 1f). Three types of signal were observed: (a) MtQ only, (b) MtQ and short lysosomal microtubule(s), and (c) MtQ and long lysosomal microtubule(s), showing that the lysosomal microtubule(s) are a dynamic structure. We then measured the cell body length and the length of the mNG::SPEF1 signal in cells with 1F1K1N (Figure 1e). There was a clear positive correlation between cell body length and SPEF1 signal length with the mNG::SPEF1 signal growing as the cell body length increased. We then measured the length of the new flagellum and mNG::SPEF1 length in cells with two flagella. In these cells, the mNG::SPEF1 signal length remained fairly constant at ~10 µm until the new flagellum reached ~4 µm in length at which point there was a dramatic shortening of the mNG::SPEF1 signal from the posterior end, which coincided with the onset of cell division. In these cells, the only mNG::SPEF1 signal remaining is that associated with the MtQ and pocket microtubules near the flagellar pocket (Figure 1d). In late cytokinesis, when the new flagellum has grown to ~7 µm, a short mNG::SPEF1 signal was observed, suggested lengthening of the lysosomal microtubule(s) as the new flagellum grew (Figure 1e).

**Figure A1:**
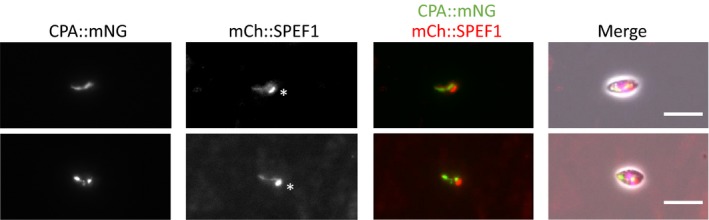
Localization of CPA::mNG and mCh::SPEF1 in axenic amastigotes. Images of CPA::mNG (green) and mCh::SPEF1 (red) colocalization in axenic amastigotes. The scale bar represents 5 µm. White asterisk in the mCh::SPEF1 channel indicates the anterior end of the cell. mCh::SPEF1 localized to two structures a bright spot close to the flagellar pocket and a fainter curved line extending toward the posterior end of the cell. Two types of CPA::mNG localization pattern were observed, either a curved line extending from near the flagellar pocket to the posterior end of the cell or a series of variable sized points that followed a line through the cell. These large spots correlate with the megasomes previously observed by TEM (Waller & McConville, 2002). Both types of CPA::mNG localization pattern run alongside the mCh::SPEF1 signal, indicating a close association of these proteins in the amastigote form

Given the parallels of localization patterns between CPA::mNG and mNG::SPEF1 during the cell cycle, we generated a cell line expressing both CPA tagged with mCherry (mCh) and SPEF1 tagged with mNG (Figure 1g). At the start of the cell cycle, the CPA::mCh‐labeled MVT lysosome lay parallel to the mNG::SPEF1 signal, consistent with mNG::SPEF1 localizing to the lysosomal microtubule(s). Through the cell cycle, these two structures have similar dynamics, extending toward the cell posterior and then disassembling before cell division. This pattern of cell cycle dynamics is similar to that of the cytostome/cytopharynx and its associated microtubules in *T. cruzi* (Alcantara et al., 2017). We differentiated the promastigote cells expressing CPA::mNG and mCh::SPEF1 in vitro to axenic amastigotes and then imaged them (Figure A1). In the axenic amastigotes, mCh::SPEF1 localized to two structures a bright spot close to the flagellar pocket and a fainter curved line extending toward the posterior end of the cell. Two types of CPA::mNG localization pattern were observed, either a curved line extending from near the flagellar pocket to the posterior end of the cell or a series of variable sized points that followed a line through the cell. These large spots correlate with the megasomes previously observed by TEM in amastigotes (Waller & McConville, 2002). Both types of CPA::mNG localization pattern run alongside the mCh::SPEF1 signal, indicating a close association of these proteins in the amastigote form.

**Figure 2:**
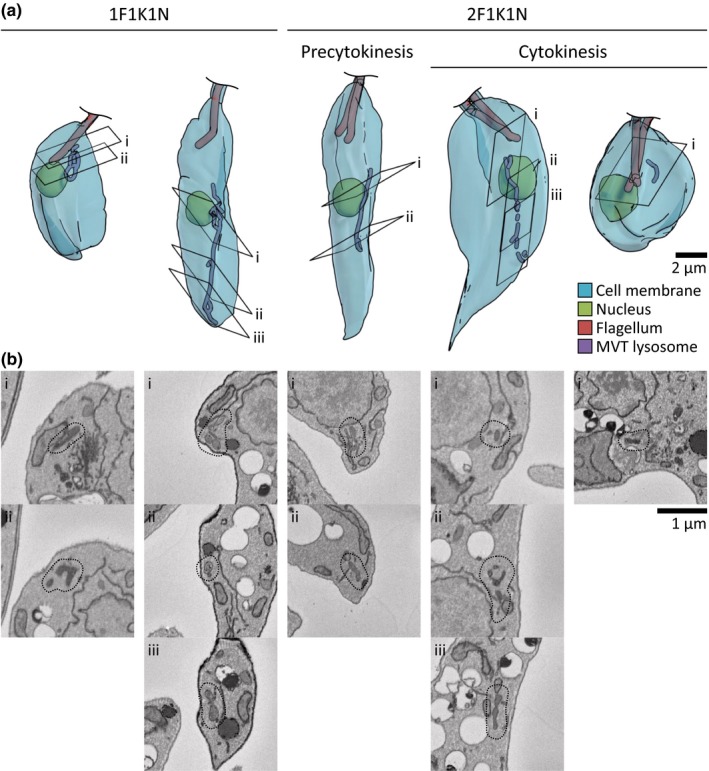
Three‐dimensional structure of the MVT lysosome throughout the cell cycle. (a) Three‐dimensional reconstructions of the nuclei (green), flagella (red), and MVT lysosomes (purple) of *Leishmania* promastigotes from serial block‐face scanning electron microscopy images. Major cell cycle stages similar to those in Figure 1 are shown. From left to right: Early cell cycle 1F1K1N, midcell cycle 1F1K1N, precytokinesis 2F1K1N, early cytokinesis 2F1K1N, and late cytokinesis 2F1K1N. (b) SBFSEM images illustrating example features of the MVT lysosome of the cells shown in (a). The images labeled i, ii, or iii correspond to the sections with the matching label in (a) and the MVT lysosome in each section is outlined

**Figure A2:**
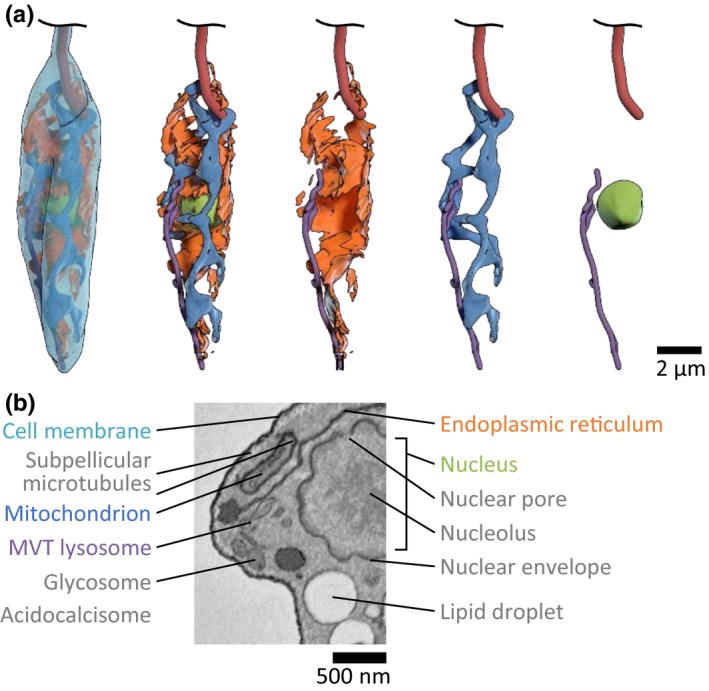
Interpretation of cell structure from scanning block‐face electron microscopy (relating to Figure 2). (a) Three‐dimensional reconstructions of the major membrane‐bound cytoplasmic structures, endoplasmic reticulum (orange), mitochondrion (blue), and MVT lysosome (purple), in an example 1K1N *Leishmania* promastigote from serial block‐face scanning electron microscopy images. The MVT lysosome is a third extended membrane‐bound network in addition to the endoplasmic reticulum and mitochondrion. (b) SBFSEM image illustrating identifiable structures in the cell. A combination of appearance (electron density) and 3D shape allow organelle identification. Acidocalcisomes and lipid droplets are near‐spherical organelles with high and low electron density, respectively. Glycosomes are more elongated with intermediate electron density. The endoplasmic reticulum, nuclear envelope, mitochondrion, and MVT lysosome form extended tubes/networks. The endoplasmic reticulum and nuclear envelope have a narrow lumen and the two membranes are not well resolved, instead appearing as a single highly electron dense line. The nucleus is identifiable from the chromatin contents. The mitochondrion lumen is well resolved with cristae occasionally visible. The double membrane is not well resolved, again appearing as a single highly electron dense line. The MVT lysosome has a wider lumen than the endoplasmic reticulum and does not have lamellar regions. The MVT lysosome is bounded by a single membrane which appears less electron dense than the mitochondrion or endoplasmic reticulum membranes

To confirm that changes in CPA::mNG localization correspond to ultrastructural changes in the MVT lysosome, we used serial block‐face scanning electron microscopy (SBFSEM) to reconstruct the MVT lysosome in three dimensions (Figure 2). A combination of appearance (electron density) and 3D shape allowed organelle identification, and in Figure A2, we have explained the criteria we used to discriminate the MVT lysosome from other tubular organelles. In SBFSEM images, the lysosomal microtubule(s) did not have sufficient contrast to be visible, and however, the MVT lysosome appears as a branching tubule around 100 nm wide. We analyzed 24 cells at random stages of the cell cycle and consistently observed MVT lysosome structures consistent with the CPA::mNG result (Figure 2)—long tubular structures with occasional branches in long (mid cell cycle) 1F1K1N cells and 2F1K1N cells prior to cytokinesis, while in small (early cell cycle) 1F1K1N and late cytokinesis cells there were no long tubular structures.

**Figure 3:**
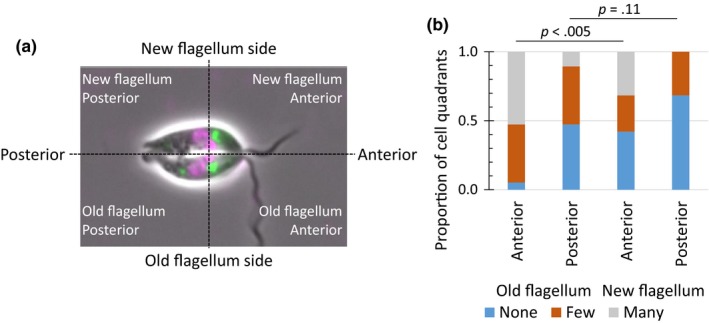
Distribution of the CPA::mNG labeled vesicles in cells undergoing cytokinesis. (a) Example of a cell undergoing cytokinesis divided into four quadrants depending on whether they were in the posterior or anterior portion of the cell which would inherit either the old or the new flagellum. (b) Bar chart showing the number (none, few, and many) of vesicles present in each quadrant. *n* = 19

To understand the mechanism of lysosome division and reassembly, we analyzed the distribution of the CPA labeled vesicles in cells undergoing cytokinesis by dividing the cell into four quadrants that corresponded to the posterior or anterior portion of the cell and the side of the cell that will become the daughter inheriting the old flagellum or the side of the cell that will inherit the new flagellum (Figure 3). Vesicles were more commonly found in the anterior old flagellum quadrant than in the equivalent anterior new flagellum quadrant. In the cell, posterior vesicles were only slightly more commonly found in the posterior old flagellum quadrant than the posterior new flagellum quadrant. There was uneven segregation of the CPA labeled vesicles with them more likely to remain associated with the old flagellum inheriting cell, suggesting there is no efficient mechanism to ensure the even distribution these vesicles. No early G_1_ cells were observed that were devoid of CPA labeled vesicles, and it is likely that these vesicles are used to nucleate the regeneration of the MVT lysosome; however, we cannot preclude that the MVT lysosome could also be generated de novo from endosomes positioned close to the flagellar pocket.

**Figure A3:**
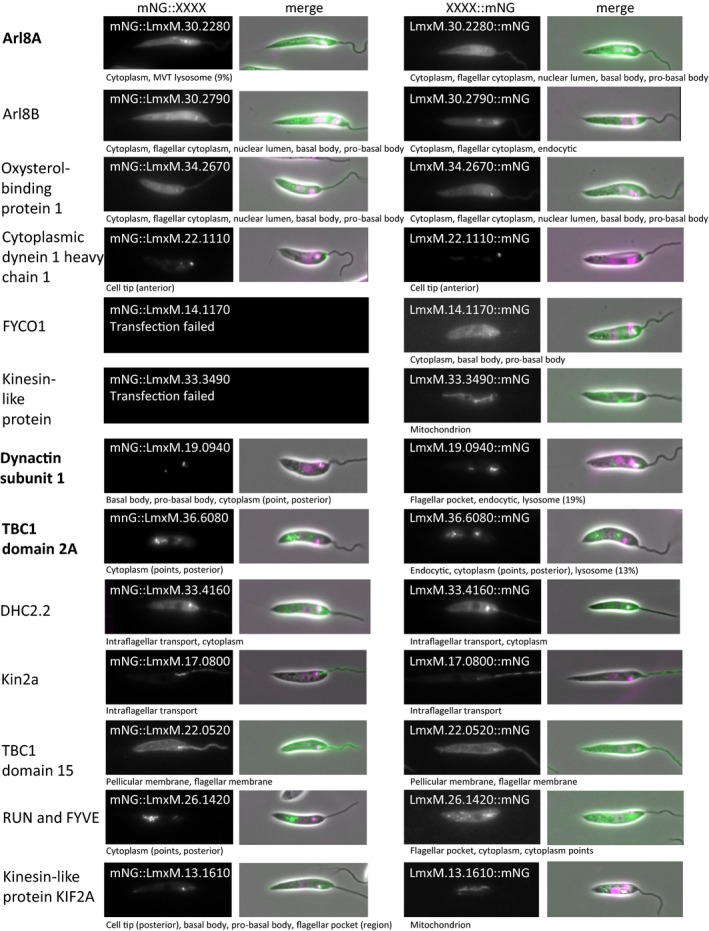
Localization of proteins identified in *L. mexicana* with identity to known regulators of lysosome function (relating to Table A1). Images of cell lines expressing identified proteins tagged at either the N‐ or C‐terminus with mNG in *Leishmania* promastigotes. Nuclear and kinetoplast DNA were labeled with Hoechst 33342. The name of the human protein is on the right with the corresponding *L. mexicana* gene ID in white text in the mNG channel image. Beneath each set of images is the description of the protein localization. The proteins in bold are those cell lines which have a proportion of cells in which a lysosome localization was observed

Lysosomes in other eukaryotes including humans are highly mobile organelles capable of moving throughout the cytoplasm (Pu, Guardia, Keren‐Kaplan, & Bonifacino, 2016). Moreover, in activated macrophages the lysosome undergoes a process called tubulation in which long lysosomal tubules are formed (Mrakovic, Kay, Furuya, Brumell, & Botelho, 2012; Swanson, Bushnell, & Silverstein, 1987). The dynamics of the lysosome in mammalian cells require microtubules and associated proteins such as kinesins and dyneins. We searched the *L. mexicana* genome for orthologs of proteins implicated in lysosomal movement using BLAST (Table A1; Pu et al., 2016). Of the 36 proteins, we interrogated the *L. mexicana* genome with only 21 had significant hits; however, only six of these were reciprocal best BLAST hits. The majority of the identified proteins were part of the kinesin or dynein complexes with many of the adaptor and effector proteins required for lysosome biogenesis and movement absent in the *L. mexicana* genome. We generated cell lines in which these bioinformatically identified proteins were endogenously tagged at either their N‐terminus or their C‐terminus and examined their localization by fluorescence microscopy (Figure A3). The majority of the proteins did not have a localization that corresponded to the MVT lysosome, except for ARL8A, dynactin, and TBC1 domain 2A protein, where in 9%, 19%, and 13% of one flagellum cells, respectively, there was a signal that corresponded to the likely position of the MVT lysosome. However, for none of these proteins was the signal along the length of the MVT lysosome, suggesting that they are unlikely to have a role in defining the lysosome‐MVT tubular structure.

### 2.2 Endocytosis rate changes during the cell cycle

**Figure 4:**
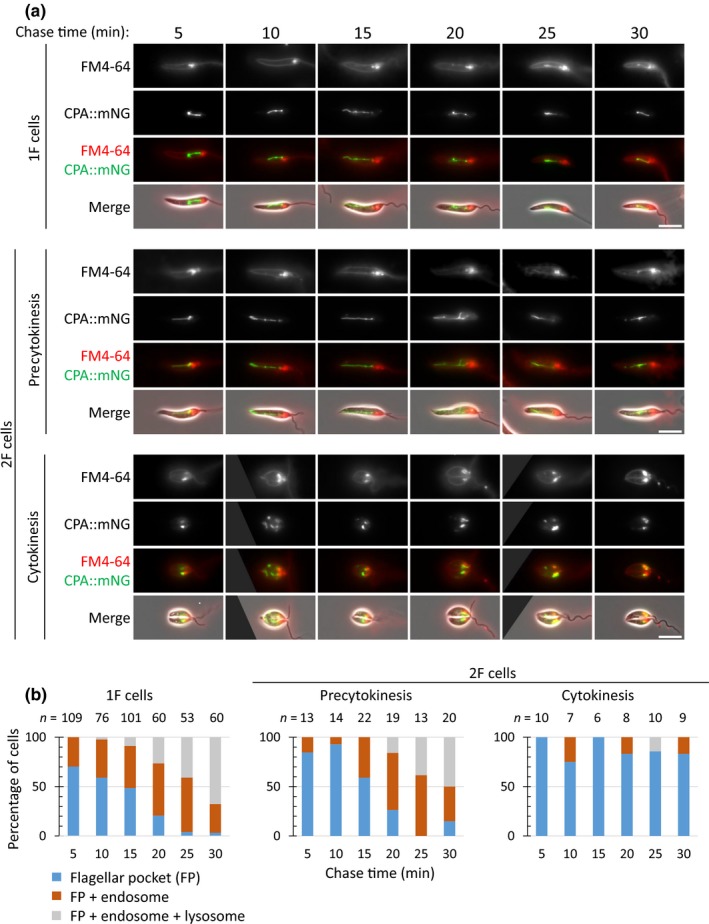
FM4‐64 pulse‐chase in the CPA::mNG cell line. (a) FM4‐64 pulse‐chase assay with promastigotes expressing CPA::mNG. Promastigotes were chilled on ice for 20 min and then pulsed with FM4‐64 for 1 min before imaging every 5 min over a 30 min time course. Three major categories of FM4‐64 localization were observed: flagellar pocket; flagellar pocket and endosome; and flagellar pocket, endosome, and lysosome. The scale bar represents 5 µm. (b) Proportion of each category observed at each time point for cells for 1F, 2F, and cells in cytokinesis. Numbers counted for each time point are indicated above the columns. The uptake assays were done independently three times, and results from a representative experiment are shown

**Figure 5:**
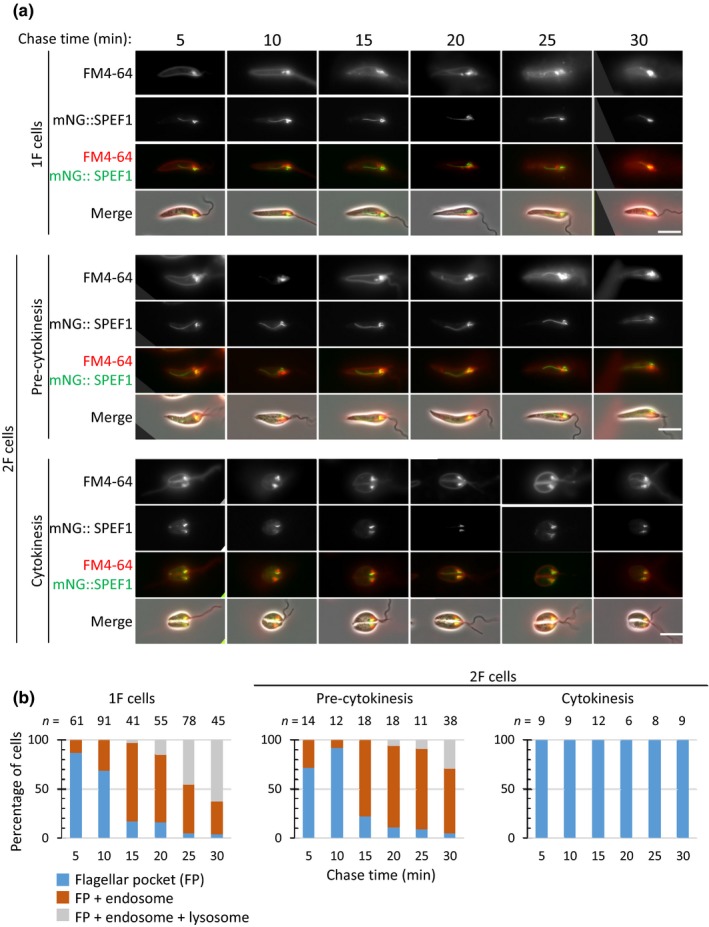
FM4‐64 pulse‐chase in the mNG::SPEF1 cell line. (a) FM4‐64 pulse‐chase assay with promastigotes expressing mNG::SPEF1. Promastigotes were chilled on ice for 20 min and then pulsed with FM4‐64 for 1 min before imaging every 5 min over a 30 min time course. Three major categories of FM4‐64 localization were observed: flagellar pocket; flagellar pocket and endosome; and flagellar pocket, endosome, and lysosome. The scale bar represents 5 µm. (b) Proportion of each category observed at each time point for cells for 1F, 2F, and cells in cytokinesis. Numbers counted for each time point are indicated above the columns. The uptake assays were done independently three times, and results from a representative experiment are shown

The MVT lysosome is the terminal endocytic compartment in *Leishmania,* and hence, we assessed the effect of disassembly of this organelle on endocytosis. We analyzed endocytosis of membrane in cell lines expressing either CPA::mNG or mNG::SPEF1 at different stages of the cell cycle using the lipophilic dye FM4‐64. The cells were chilled on ice and then given a short pulse of FM4‐64, and the cells were then imaged at 5‐min intervals over a 30‐min time course. Both cell lines behaved in a similar manner (Figures 4 and 5). In cells with one flagellum, FM4‐64 was initially found on the cell membrane and within the flagellar pocket (Figures 4 and 5). As the time course progressed, the signal was observed in the endosome before finally labeling the MVT lysosome, where it colocalized with the CPA::mNG signal and was parallel to the mNG::SPEF1 signal. Over the time course, the number of cells with a MVT lysosome signal steadily increased, showing movement from the flagellar pocket through the endocytic system (Figures 4 and 5).

Next, we analyzed cells with two flagella before they entered cytokinesis (Figures 4 and 5). The situation here was more complicated as some of these cells are in the process of disassembling their MVT lysosome. Over the time course, FM4‐64 was able to progress from the cell membrane and flagellar pocket into the endosome and finally the MVT lysosome (Figures 3 and 4). However, there was generally a lower proportion of cells with a MVT lysosome signal at the 30‐min time point than seen for the cells with one flagellum. In those cells in which the MVT lysosome had fully disassembled, the FM4‐64 signal was never observed beyond the flagellar pocket region and this population of cells is likely to account for the reduced proportion seen with a MVT lysosome signal.

In cells undergoing cytokinesis in which the CPA and SPEF1 signal associated with the MVT lysosome had disassembled, the FM4‐64 dye was initially observed on the cell membrane and in the flagellar pocket as with the other cell cycle stages (Figures 4 and 5). However, as the time course continued, FM4‐64 did not generally progress to later endocytic compartments and remained associated with the flagellar pocket (Figures 4 and 5). Even at later time points when the majority of cells with one flagellum had a MVT lysosome signal, the signal in cells undergoing cytokinesis was still restricted to the flagellar pocket. In the cell line expressing CPA::mNG, a few cells were categorized as having a FM4‐64 lysosome signal; however, this is potentially due to the remaining CPA::mNG signal in the flagellar pocket region overlapping with the FM4‐64 in that area (Figure 4). Moreover, this cell type was not observed in the cells expressing mNG::SPEF1, and as at this stage, the mNG::SPEF1 lysosomal microtubule signal was completely absent, eliminating the chance of overlap (Figure 5).

**Figure A4:**
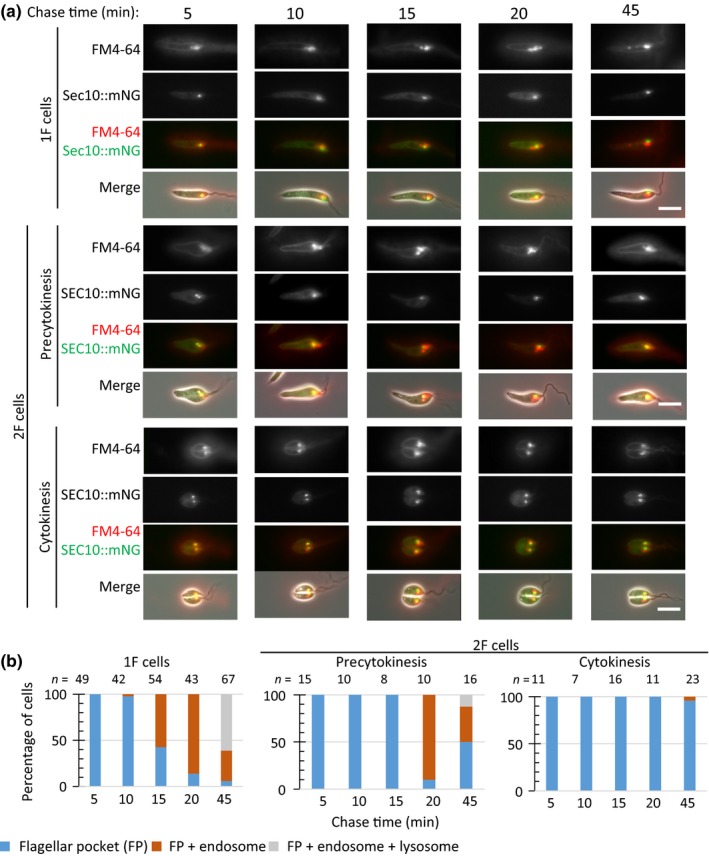
FM4‐64 pulse‐chase in the SEC10::mNG cell line. (a) FM4‐64 pulse‐chase assay with promastigotes expressing SEC10::mNG. Promastigotes were chilled on ice for 20 min and then pulsed with FM4‐64 for 1 min before imaging over a 45‐min time course. Three major categories of FM4‐64 localization were observed: flagellar pocket; flagellar pocket and endosome; and flagellar pocket, endosome, and lysosome. The scale bar represents 5 µm. (b) Proportion of each category observed at each time point for cells for 1F, 2F, and cells in cytokinesis. Numbers counted for each time point are indicated above the columns. The uptake assays were done independently three times, and results from a representative experiment are shown

To determine precisely where FM4‐64 accumulates in dividing cells, we investigated endocytosis further in a cell line expressing SEC10::mNG, which we have previously used as a marker of the flagellar pocket (Sunter et al., 2019). As expected in cells with one or two flagella not undergoing cytokinesis, FM4‐64 was initially found on the cell membrane and within the flagellar pocket (Figure A4). As the time course progressed, the signal was observed in the endosome before finally labeling the MVT lysosome. In cells undergoing cytokinesis in which the MVT lysosome would have disassembled, FM4‐64 was initially observed on the cell membrane and in the flagellar pocket as with the other cell cycle stages (Figure A4). However, as the time course continued, FM4‐64 did not progress to later endocytic compartments and remained associated with the flagellar pocket region as shown by its colocalization with SEC10 (Figure A4). However, the colocalization of SEC10::mNG and FM4‐64 was not complete, suggesting that the FM4‐64 was internalized into the endocytic system surrounding the flagellar pocket but was not able to progress any further. Together, these data indicate that the rate of endocytosis in cells undergoing cytokinesis is greatly reduced.

## 3 CONCLUSIONS

In many eukaryotes, the disassembly of organelles preceding cell division followed by their reassembly in the subsequent cell cycle is commonly observed; however, kinetoplastids have a different pattern with many organelles duplicated through the cell cycle then segregated. Here, we show that the MVT lysosome and lysosomal microtubule(s) in *Leishmania* are an exception to this pattern as they were disassembled in late G_2_ prior to cell division before being reassembled during the next G_1_ phase. The requirement for the MVT lysosome and associated microtubule(s) to disassemble before cell division suggests that their continued presence would impede cell division in some way.

The anterior end of the MVT lysosome structure is intimately linked to the flagellar pocket via the site of nucleation of the lysosomal microtubule(s). As mNG:SPEF1 signal always extended from the pocket, while CPA::mNG signal often started further into the cell, this provides further evidence that the lysosomal microtubule(s) could be a mechanical support for the MVT lysosome. The intimate linkage of the lysosomal microtubule(s) with the flagellar pocket suggests that the MVT lysosome may disassemble to facilitate or enable flagellar pocket division. It is also possible that the disassembly of the existing MVT lysosome is required to facilitate assembly alongside the new lysosomal microtubule(s). This raises the issue of dependency relationships between the MVT lysosome and the lysosomal microtubule(s) and whether assembly of the MVT lysosome requires the lysosomal microtubule(s) or vice versa. It is tempting to speculate that the MVT lysosome would follow the path of the assembling lysosomal microtubule(s); however, we have no evidence to support this idea. To test the dependence of lysosome morphology on SPEF1, we attempted to generate a SPEF1 null mutant in *L. mexicana* on several occasions but were never successful, suggesting that SPEF1 is an essential protein. *T. brucei* lacks lysosomal microtubule(s), and the lysosome in *T. brucei* has a different architecture—although prior investigation of the phenotype of SPEF1 depletion in *T. brucei* did not analyze whether there was an effect on the lysosome (Gheiratmand et al., 2013).

During our search for lysosomal protein markers (Halliday et al., 2019), we noticed that the lysosomal protein p67 found in *T. brucei* and *T. cruzi*, which is related to the lysosome‐associated membrane proteins found in the lysosome of many eukaryotes, is missing from the *Leishmania* genomes. In *T. brucei,* p67 has a function in maintaining the morphology of the lysosome and the loss of p67 in *Leishmania* might be associated with the unusual structure of the MVT lysosome (Peck et al., 2008).

The dynamic nature of the MVT lysosome has striking parallels with cytostome/cytopharynx of *T. cruzi*, which also undergoes a similar cell cycle regulation, disassembling before cytokinesis, and reassembling afterward (Alcantara et al., 2017). Moreover, the MVT lysosome and the cytostome/cytopharynx have a similar membrane tubule structure with associated microtubules which nucleate near the flagellar pocket. The cytostome/cytopharynx was likely an ancestral feature of kinetoplastids and given the similar cell cycle dynamics and overall architecture perhaps the MVT lysosome and lysosomal microtubule(s) are a reduced cytostome/cytopharynx‐like feature (Skalický et al., 2017; Weise et al., 2000). However, there are significant differences in the orientation and path of the cytosome/cytopharynx and lysosomal microtubule(s). We also found that when the MVT lysosome has disassembled, there was a dramatic reduction in the rate of endocytosis, which again was observed when the cytostome/cytopharynx disassembled in *T. cruzi* (Alcantara et al., 2017).

Here, we have provided insight into the cell cycle‐dependent restructuring of the late endocytic system and the resulting effect on endocytic rate in *Leishmania*. The disassembly of the MVT lysosome is likely to be a critical step in *Leishmania* cell division and as such deciphering the regulation of this process within the context of the cell cycle will be an important step in understanding cell cycle coordination in these organisms. Our study highlights further commonalities and differences between the “TriTryps” and reinforces the added value that can be gained from comparative analyses of basic cell processes between the different kinetoplastids.

## 4 Experimental procedures

### 4.1 Cell culture

Cas9T7 *L. mexicana* (derived from WHO strain MNYC/BZ/62/M379, expressing Cas9 and T7 RNA polymerase) promastigotes were grown in M199 medium with Earle's salts, and L‐glutamine supplemented with 10% (v/v) heat‐inactivated FCS, 5 mM HEPES‐NaOH (pH 7.4), 26 mM NaHCO_3_, and 5 μg/ml haemin at 28°C. Axenic amastigotes were generated by subculture into Schneider's Drosophila medium with 20% heat‐inactivated FCS and 25 mM MES‐HCL (pH 5.5) at 34°C with 5% CO_2_ for 72 hr.

### 4.2 Generation of tagging constructs

Generation of the *L. mexicana* tagging constructs and sgRNA templates for endogenous mNeonGreen or mCherry tagging were generated by the PCR method as previously described using pLPOT (mNG/Blasticidin) or pLPOT (mCh/Puromycin) as the template, respectively (Halliday et al., 2019). Transfection of cells was performed as previously described using the Amaxa Nucleofector‐2b (Dean et al., 2015). Primers for constructs and sgRNA were designed using LeishGEdit (http://www.leishGEdit.net). Successful transfectants were selected with 5 μg/ml Blasticidin S hydrochloride (Melford Laboratories) or 20 μg/ml Puromycin (Melford Laboratories) 6 hr following transfection.

### 4.3 Fluorescence microscopy and morphometric measurements

For live cell microscopy, cells were harvested by centrifugation at 800 *g* for 5 min and washed three times in PBS with Hoechst 33342 (1 μg/ml) in the first wash. The cells were resuspended in 30 μl PBS, and 1 μl was then placed on a microscope slide and immediately imaged using a Zeiss ImagerZ2 microscope with a 63 × NA 1.4 objective and Hamamatsu Flash 4 camera. Length measurements were made in ImageJ (Rueden et al., 2017).

### 4.4 Pulse‐chase endocytosis assay

Promastigotes (5 × 10^6^ cells) were incubated in complete M199 medium on ice for 20 min before 40 μg/ml FM4‐64 (Invitrogen) was added for 1 min. Cells were immediately harvested by centrifugation at 800 *g* and resuspended in prewarmed M199 with no dye at 28°C. At each time point, cells were removed and washed with PBS before imaging.

### 4.5 SBFSEM

*Leishmania mexicana* cells were fixed in culture with a final concentration of 2.5% glutaraldehyde for 2 min. The cells were then pelleted at 800 g for 3 min and resuspended in 100 mM phosphate buffer pH 7.4, containing 2.5% glutaraldehyde and 2% formaldehyde. The pellet was washed with 100 mM phosphate buffer pH 7.4 and then postfixed in 1% osmium tetroxide and 1.5% potassium ferrocyanide in 100 mM phosphate buffer pH 7.4 buffer for 1 hr. The sample was rinsed in ddH_2_O and then incubated in 1% thiocarbohydrazide for 20 min. The sample was rinsed in ddH_2_O and then incubated 2% osmium tetroxide for 30 min. After rinsing, the sample was stained overnight in 2% uranyl acetate at 4°C. The sample was then rinsed again then dehydrated in an ascending ethanol series and embedded in TAAB 812 hard resin (TAAB Laboratories Equipment Ltd). The block was trimmed and placed into a Zeiss Merlin VP Compact fitted with a Gatan 3view2XP system. Serial images of the block face were recorded at an accelerating voltage of 1.5 kV, a spot size of 1 and an aperture size of 20 μm, and pressure of 0.0 Torr. Pixel size and the dwell time for each micrograph was 2 nm, 1 μs, and slice thickness was 75 nm. Images were recorded using Digital Micrograph. Three‐dimensional models were generated by tracing the SBFSEM images using IMOD (Kremer, Mastronarde, & McIntosh, 1996) and visualized using Blender. MVT lysosomes were distinguishable from glycosomes due to higher luminal electron density and greater length and distinguishable from mitochondrion branches due to lower membrane electron density than the double mitochondrial membrane.

## CONFLICT OF INTEREST

The authors declare no conflict of interest.

## AUTHOR CONTRIBUTIONS

Conceptualisation: RJW, JDS

Formal analysis: ZW, RJW, JDS

Funding investigation: RJW

Methodology: RJW, JDS

Project administration: JDS

Visualisation: ZW, RJW, JDS

Writing – original draft: ZW, RJW, JDS

Writing – review & editing: RJW, JDS

## ETHICS STATEMENT

None required.

## Data Availability

All data are provided in full in the results section of this paper.

## Appendix 1

**Table A1:** Human lysosomal proteins identified from Pu et al., 2016, against *Leishmania mexicana* genome

Protein name	Uniprot ID	Blast E‐score	*L. mexicana* ID	Blast E‐score	*T. brucei* ID	Ortholog to *L. mexicana*
Kinesin−1 heavy chain (KIF5B)	P33176	3.00E−74	LmxM.17.0800	5.00E−89	**Tb927.1.1350**	No
Kinesin‐like protein (KIF3A)	Q9Y496	2.00E−100	**LmxM.17.0800**	2.00E−102	**Tb927.5.2090**	Yes
Kinesin‐like protein (KIF3B)	O15066	2.00E−98	LmxM.17.0800	1.00E−107	Tb927.5.2090	Yes
Kinesin‐like protein (KIF1A)	Q12756	1.00E−95	LmxM.33.4260	2.00E−93	Tb927.11.2490	No
Kinesin‐like protein (KIF1B beta)	O60333	2.00E−94	LmxM.33.4260	2.00E−88	Tb927.11.2490	No
Kinesin‐like protein (KIF5A)	Q12840	1.00E−72	LmxM.17.0800	2.00E−84	Tb927.1.1350	No
Kinesin‐like protein (KIF5C)	O60282	4.00E−72	LmxM.17.0800	3.00E−89	Tb927.1.1350	No
Kinesin‐like protein (KIF2A beta)	O00139	2.00E−106	LmxM.13.1610	7.00E−110	Tb927.9.3650	No
Protrudin	Q5T4F4	NO HIT		3.00E−05	Tb927.7.3790	
Kinesin‐like chain 1 (KLC1)	Q07866	7.00E−12	**LmxM.33.3490**	4.00E−14	Tb927.4.1280	Yes
KLC2	Q9H0B6	1.00E−09	LmxM.33.3490	4.00E−12	Tb927.4.1280	Yes
KLC3	Q6P597	2.00E−06	LmxM.33.3490	2.00E−08	Tb927.4.1280	Yes
KLC4	Q9NSK0	2.00E−09	LmxM.33.3490	2.00E−10	Tb927.4.1280	Yes
Biogenesis of lysosome‐related organelles complex 1 subunit1 (BLOS1)	P78537	NO HIT				
BLOS2	Q6QNY1	NO HIT				
SNARE‐associated protein	O95295	NO HIT				
KXD1	Q9BQD3	NO HIT				
BLOS8	Q96FH0	NO HIT				
BORCS5	Q969J3	NO HIT				
Lyspersin	Q96GS4	NO HIT				
Diaskedin	Q96B45	NO HIT				
ADP‐RIBOSYLATION FACTOR‐LIKE PROTEIN 8B (Arl8B)	Q9NVJ2	1.00E−32	**LmxM.30.2790**	3.00E−33	Tb927.9.13650	Yes
ADP‐RIBOSYLATION FACTOR‐LIKE PROTEIN 8A (Arl8A)	Q96BM9	1.00E−30	LmxM.30.2280	2.00E−31	Tb927.10.8580	No
SifA	Q8IWE5	NO HIT				
FYCO1	Q9BQS8	1.00E−10	LmxM.14.1170	1.00E−09	Tb927.7.3790	Yes
Rab‐interacting lysosomal protein (RILP)	Q96NA2	NO HIT				
Oxysterol‐binding protein‐related protein 1	Q9BXW6	1.00E−13	LmxM.34.2670	1.00E−12	Tb927.4.1170	No
Dynactin subunit 1	Q14203	4.00E−11	**LmxM.19.0940**	5.00E−10	Tb927.10.14770	No
Dynactin subunit 2	Q13561	NO HIT				
TBC1 domain family member 15	Q8TC07	5.00E−38	**LmxM.22.0520**	2.00E−43	Tb927.7.2470	Yes
TBC1 domain family member 2A	Q9BYX2	3.00E−34	LmxM.36.6080	8.00E−31	Tb927.10.8750	Yes
RUN and FYVE	Q96T51	6.00E−09	LmxM.26.1420	9.00E−14	Tb927.7.1840	No
Mon1–Ccz1	Q96DM3	NO HIT		8.00E−04	**Tb927.7.6650**	
Cytoplasmic dynein 1 heavy chain 1	Q14204	0	**LmxM.22.1110***	0	Tb927.4.560	Yes
Lysosome‐associated membrane glycoprotein−1 (LAMP−1)	P11279	NO HIT				
LAMP−2	P13473	NO HIT				

Note

Sequences were obtained from Uniprot (https://www.uniprot.org), and entries for UniProt proteins were obtained from UniProtKB release. Blast searches were performed using UniProt Blast against Fasta formatted databases (UniProt or UniProt/Swiss‐Prot). Cutoff for E‐score is less than 10^−5^. Proteins in bold were identified via reciprocal Blast. *Leishmania* Cytoplasmic dynein 1 heavy chain 1 protein was identified from Wickstead & Gull, 2007.
